# Estimating HIV Prevalence in Zimbabwe Using Population-Based Survey Data

**DOI:** 10.1371/journal.pone.0140896

**Published:** 2015-12-01

**Authors:** Amos Chinomona, Henry Godwell Mwambi

**Affiliations:** 1 Department of Statistics, Rhodes University, Grahamstown, South Africa; 2 School of Mathematics, Statistics and Computer Science, University of Kwa-Zulu Natal, Pietermaritzburg, South Africa; Alberta Provincial Laboratory for Public Health/ University of Alberta, CANADA

## Abstract

Estimates of HIV prevalence computed using data obtained from sampling a subgroup of the national population may lack the representativeness of all the relevant domains of the population. These estimates are often computed on the assumption that HIV prevalence is uniform across all domains of the population. Use of appropriate statistical methods together with population-based survey data can enhance better estimation of national and subgroup level HIV prevalence and can provide improved explanations of the variation in HIV prevalence across different domains of the population. In this study we computed design-consistent estimates of HIV prevalence, and their respective 95% confidence intervals at both the national and subgroup levels. In addition, we provided a multivariable survey logistic regression model from a generalized linear modelling perspective for explaining the variation in HIV prevalence using demographic, socio-economic, socio-cultural and behavioural factors. Essentially, this study borrows from the proximate determinants conceptual framework which provides guiding principles upon which socio-economic and socio-cultural variables affect HIV prevalence through biological behavioural factors. We utilize the 2010–11 Zimbabwe Demographic and Health Survey (2010–11 ZDHS) data (which are population based) to estimate HIV prevalence in different categories of the population and for constructing the logistic regression model. It was established that HIV prevalence varies greatly with age, gender, marital status, place of residence, literacy level, belief on whether condom use can reduce the risk of contracting HIV and level of recent sexual activity whereas there was no marked variation in HIV prevalence with social status (measured using a wealth index), method of contraceptive and an individual’s level of education.

## Introduction

Zimbabwe ranks among the countries that have been worst affected by the HIV and AIDS epidemic in sub-Saharan Africa. Although studies have shown that HIV prevalence has been falling since the late 90s, an estimate from the National HIV estimates of 2010, of approximately 15% among the country's sexually active population (15 years and above) is still considered to be too high. The factors that have contributed to the observed decline in HIV prevalence since the late 90s include robust prevention programs, significant behaviour change that has reduced new infections and the successful implementation of the Prevention of Mother-to-Child Transmission (PMTCT) program. Recent studies have also revealed that the relatively high literacy level among the country's population has allowed rapid and effective dissemination of information on HIV and AIDS awareness and appropriate preventive measures, [1], [2]. These have in turn resulted in increased and consistent use of condoms, reduction in casual sex, reduction in extramarital partners and in commercial sex activities.

There is a wealth of literature on HIV/AIDS in Zimbabwe based on surveillance data. These range from Antenatal Clinic (ANC) surveys ran since 1990, Behavioural data obtained from the 2001/2002 Young Adult Survey (YAS), the 2001 and 2003 Population Services International (PSI) Youth Surveys, the Zimbabwe Demographic Health Surveys (1988, 1994, 1999 and 2005/6 with the latest one being 2010–2011), the World Health Organization (WHO) and the UNAIDS. Zimbabwe's HIV epidemic, just like in other sub-Saharan African countries, is mainly driven by heterosexual transmission, [3]. This has mainly been linked to networks of multiple sexual relations, including concurrent relations in which the virus is passed on rapidly. The current socio-economic and socio-political situations in the country have also exacerbated the spread of the virus. Despite aggressive awareness programs, studies have shown that increased commercial sex activities resulting from high unemployment rates have contributed significantly to new cases and the spread of the virus. Studies have also established that a leading cause of HIV infections in new-born babies is the MTCT, [4]. The 2001 National HIV Sentinel Surveillance Survey estimated that an antenatal nationwide HIV sero-prevalence of 29.5% acquire HIV from their mothers annually in Zimbabwe [5]. The authors in [6] reported that MTCT before, during and after delivery may result in the acquisition of HIV for 30–35% of infants of HIV-infected mothers.

Statistical methods have been widely used to assess the trends, patterns and the dynamics of HIV/AIDS epidemic. The focus of these methods range, depending on the objectives embedded in the research, from estimating prevalence, understanding the pathogenesis of HIV infection, assessing the potency of antiviral therapies and evaluation of treatment efficacy of viral dynamic models, [7]. Statistical models that estimate the magnitude and trajectory of the HIV/AIDS epidemic have also become necessary. These models have been used as tools to extract and convey as much information as possible from available data and provide accurate representation of both the knowledge (understanding origin and progression) and uncertainty about the epidemic. The success of the statistical methods used relies on the quality of the data available. In addition, the quality and reliability of the estimates calculated from the available data depends on the validity of the statistical analysis performed. Sampling and survey (including complex surveys) techniques have been used as bases for accurate data collection and provide sufficient theory behind consistent analysis of phenomena such as HIV epidemic. A key role of the discipline of statistics is to provide science with theory and methods for objective evaluation of data-based evidence and measuring the strength of that evidence, [8].

Previous studies involving national HIV prevalence estimation have mainly been based on data obtained from subgroups of the population. For instance, data obtained from pregnant women attending ANCs, from blood donors, from truck drivers, from prison populations, from commercial sex workers, from men who sleep with other men and from drug users, see [1], [7], [9]. The representativeness of these data to the target population has been argued to be inadequate, see [10]. The current study investigates how and describes the way in which HIV prevalence varies with demographic, socio-economic, socio-cultural and behavioural risk factors using population-based DHS data. We consider the extent to which the association between HIV status is affected by a person's age, gender and marital status, education, place of residence, wealth status, religion and behaviour towards HIV. The study exploits the strength of statistical methods to provide estimates of HIV prevalence at the national and domain (subgroup) levels using nationally representative sample survey data. In addition, from a statistical modelling approach, a multivariable survey logistic regression model was computed.

## Materials and Methods

### 1.1 The data

The data used for the research were obtained from the 2010–11 Zimbabwe Demographic and Health Surveys (2010–11 ZDHS). The DHSs in general are country-level population-based household surveys. The data from DHS are mainly aimed at providing information for monitoring and impact evaluation of key indicators pertaining to population, health and nutrition. Household data regarding socio-economic, health and demographic variables are collected using questionnaire-based interviews. Specifically, for the 2010–11 ZDHS females aged 15 to 49 and males aged 15 to 54 were eligible for interview and collection of blood samples or specimens, using dried blood spot (DBS) for laboratory testing (which includes HIV testing). The data used were obtained from DHS Data Archives [11].

For HIV testing in particular, blood samples were collected on a special filter paper card using capillary blood from a finger prick. An “anonymized” antibody testing process was conducted at the National Microbiology Reference Laboratory (NMRL) in Harare. Bar coded labels were used to identify the DBS samples to ensure the anonymity and these were used to track the outcome of the testing procedure and the results. Laboratory testing of the blood specimens followed a standard laboratory algorithm designed to maximize the sensitivity and specificity of the test results. In particular, the algorithm uses two different HIV antibody enzyme-linked immunosorbent assays (ELISAs) that are based on antigens. Discordant samples that were positive in the first test were retested using both ELISAs and discordant samples from the second round of testing were regarded as “indeterminate”. The “indeterminate” were then subjected to a western blot confirmatory test, in which the results were considered final. Written consent was sought from the respondents before the collection of the blood samples, and for the 15–17 year old respondents further consent was sought from their parents or responsible adult. Furthermore, consent was also sought to store blood samples for future research. All participants were given information brochures pertaining to HIV/AIDS and giving details of the nearest facility providing voluntary counseling and testing (VCT). All HIV testing procedures were reviewed and approved by the ethical review boards of ORC Macro, a US-based company that provides technical assistance to DHS worldwide, the Centers for Disease Control (CDC) and the Medical Research Council of Zimbabwe (MRCZ).

For administration purposes, Zimbabwe is divided into ten provinces. During the 2002 population census (which was used as the sampling frame in the 2010–11 ZDHS) each province was subdivided into districts and each district is made up of wards and the wards consist of a number of enumeration areas (EAs). For the current research the response variable is HIV status, a binary variable indicating whether a respondent is HIV positive or negative. The study investigates the relationship between HIV and socio-economic, socio-cultural, demographic and behavioural factors of the population using a multivariable survey logistic regression model. In determining the risk factors, the study borrows from the proximate-determinants conceptual framework as explained in [12]. Essentially, the underlying socio-economic, socio-cultural and environmental determinants operate through the proximate-determinants in order to affect an outcome such as HIV status. These factors include age, gender, marital status, education level, literacy level, economic status (wealth index), religion, province, method of contraceptive used, belief whether condom use works to reduce risk of HIV and recent sexual activities (measured in how sexually active a respondent has been in the previous four weeks) and place of residence (whether rural or urban). The sample consists of 17 434 respondents, 14 491 with non-missing values and an additional 2943 with missing values for at least one measured variable. The current study assumed a complete case analysis where we took a list-wise deletion of cases with missing values as explained in [13]. However the assumption of missing values being missing completely at random (MCAR) may be too restrictive. Future analyses may therefore need methods that correct for the impact of the missing values.

### 1.2 Statistical computing

All the analyses were done using survey package by [14] in **R** [15]. In particular, all the design features such as stratification, clustering and weighting were accounted for explicitly using the **svydesign** function. The function **svyglm** was used to describe the model by specifying the predictors and their functional form together with the link function. For automated model selection and multi-model inference, we utilized the **glmulti** package by [16], and **stepPlr** package by [17] respectively. The packages function by considering all possible explanatory variables and builds unique models for the main effects and (optionally) the pairwise interactions. A stepwise, forward selection backward elimination procedure was used to select best predictor variables. This was done by utilizing the fundamental statistical modelling framework explained in Subsection 1.3 below. The model goodness of fit was done using **ResourceSelection** package by [18]. The function **hoslem.test** was used to perform the Hosmer-Lemeshaw (H-L) test for goodness of fit as explained by [19].

### 1.3 Statistical Methods

We utilized techniques for analyzing complex survey data to compute design-consistent national and domain-level estimates of HIV prevalence and their respective significance test statistics. In particular, point estimates in the form of proportions and 100(1 − *α*)% confidence intervals were computed. The Taylor series linearization variance estimation method as explained by [20], [21], that takes the design features into account was used. In addition, design-consistent crude odds ratios (ORs) were computed to express the likelihood or the risk of being HIV for each domain in every factor.

We also computed a multivariable survey logistic regression model for explaining the variation in HIV taking the complex sampling design features into account as explained in [22]. The logistic regression models fall into a broader class of models referred to as generalized linear models (GLMs). GLMs, as first introduced by [23] and further modified by [24], are a flexible and unified class of models that are applicable to diverse types of response variables found in both normal and non-normal data.

As given in [24], a GLM consists of three components; the random component, the systematic component and the link function. Essentially, the random component is the response variable, such as HIV status (for the current study) and its probability distribution. The probability distribution has to be a member of the exponential family of distributions, see [24], [25]. The systematic component represents the predictors (the demographic, socio-economic, socio-cultural and behavioural factors) whereas the link function links the random and the systematic components. Under the conventional GLM framework the maximum likelihood estimates for the parameters are obtained via the iterative least squares method utilizing the numerical integration procedures such as the Newton-Raphson and the Fisher’s scoring. However under a complex sampling design such the one used for the 2010–11 ZDHS, a pseudo-maximum likelihood method for parameter estimation that takes the complex sampling design into account as explained in [26] is used.

Wald tests are employed to test the null hypothesis that a single coefficient is equal to zero whereas confidence intervals are further used to provide information on the potential magnitude and uncertainty associated with the estimated effects of individual predictor variables. Alternatively, the significance of predictors can be carried out directly on the log-odds scale. That is, in a logistic regression model with a single predictor, an estimate of the crude OR corresponding to a unit increase in the value of the predictor can be obtained by exponentiating the estimated logistic regression coefficient. In a multivariable logistic regression model, adjusted ORs represent the multiplicative impact of a one-unit increase in a given predictor variable on the odds of the outcome variable being equal to one, controlling for the effects of the other variables. We used the design-consistent *χ* ^2^—based H-L test proposed by [19] to assess the model goodness of fit.

## Results

We present the design-consistent descriptive estimates of HIV prevalence and their respective 95% confidence intervals at both national and domain levels. In addition, for analytic purposes a multivariable survey logistic regression model for HIV on the demographic, socio-economic, socio-cultural and behavioural factors was computed. Parameter estimates were expressed on the OR scale in order to facilitate interpretation of the logistic regression model. In particular, both the adjusted (for the effects of the other covariates in the model) and unadjusted (crude) ORs are displayed together with their respective 95% confidence intervals. It is worth pointing out that the crude descriptive estimates presented here differ slightly from those reported in the 2010–11 ZDHS report due to a number of factors. These factors may include the way in which the complex sampling design features are accounted for, the method used to handle missing data and the statistical software used.

### 2.1 Descriptive Analysis

The estimated overall design-consistent HIV prevalence in the entire population was found to be $\hat{p}=15.7\%,$ 95% *CI* = 14.7–16.0%. The prevalence estimate is close to the $\hat{p}=15.2\%$ 95% *CI* = 14.3–16.1% reported in the 2010–11 ZDHS report. The 95% CIs overlap showing that the difference is not statistically significant. HIV prevalence is known to vary considerably across population subgroups, hence in order to enhance the estimation, we computed domain level estimates of HIV prevalence. The domains considered were based on the risk factors, as informed by the proximate determinant conceptual framework as explained in [12], such as gender, marital status that form the natural subgroups of the population. The crude design-consistent estimates for the different prominent subgroups of the population are given in Table 1. Specifically, for gender the results show that the females have a higher HIV prevalence rate ($\hat{p}=17.7\%,$ 95% *CI* = 16.6–18.7%) than the males ($\hat{p}=12.8\%,$ 95% *CI* = 11.8–13.7%).

**Table 1:** Crude design-based subgroup estimates of HIV prevalence along with their respective (crude) odds ratios and 95% confidence intervals.

Risk Factor	Level	*n*	Percentages	Estimate^3^(%)	95% CI	OR	95% CI
Gender	Female	8 169	56.4	17.7	(16.6, 18.7)	Ref	
	Male	6 322	43.6	12.8	(11.8, 13.7)	0.690	(0.624, 0.763)
Marital Status	Single	4 777	33.0	5.6	(5.2, 6.6)	Ref	
	Married	8 226	56.8	16.7	(15.8, 17.7)	3.224	(2.796, 3.718)
	Divorced	877	6.1	28.8	(25.8, 32.3)	6.511	(5.320, 7.967)
	Widowed	611	4.2	54.4	(51.3, 60.0)	19.94	(16.038, 24.791)
Age group	15–19	3 295	22.7	4.0	(3.2, 4.7)	Ref	
	20–24	2 749	19.0	7.9	(6.8, 8.9)	2.109	(1.664, 2.673)
	25–29	2 522	17.4	15.8	(14.2, 17.3)	4.360	(3.495, 5.439)
	30–34	1 961	13.5	23.2	(21.3, 25.2)	7.159	(5.756, 8.902)
	35–39	1 601	11.0	26.9	(24.5, 29.2)	8.556	(6.848, 10.690)
	40–44	1 138	7.9	25.5	(22.8, 28.3)	7.865	(6.210, 9.959)
	45–49	892	6.2	25.8	(22.7, 28.8)	8.427	(6.579, 10.793)
	50–54	333	2.3	18.7	(14.3, 23.1)	5.572	(3.919, 7.921)
Place of residence Employment status Literacy^1^	Rural	9 839	67.9	14.7	(13.9, 15.4)	Ref	
	Urban	4 652	32.1	16.8	(15.7, 18.0)	1.184	(1.069, 1.312)
	Unemployed	7 900	54.5	13.5	(12.8, 14.3)	Ref	
	Employed	6 591	45.5	17.3	(16.3, 18.3)	1.350	(1.225, 1.487)
	Non-literate	859	5.9	13.9	(11.5, 16.2)	Ref	
	Partially	1 103	7.6	19.8	(17.2, 22.3)	1.489	(1.153, 1.924)
	Literate	12 529	86.5	15.1	(14.4, 15.7)	1.083	(0.884, 1.326)
Wealth Index^2^	Poorest	2 811	19.4	15.8	(14.3, 17.2)	Ref	
	Poorer	2 652	18.3	14.6	(13.2, 16.1)	0.930	(0.795, 1.086)
	Middle	2 742	18.9	16.3	(14.9, 17.9)	1.075	(0.922, 1.254)
	Richer	3 134	21.6	16.0	(14.6, 17.4)	1.055	(0.911, 1.223)
	Richest	3 152	21.8	13.9	(12.6, 15.2)	0.859	(0.738, 1.001)
Education level	No education	271	1.9	17.0	(12.3, 21.7)	Ref	()
	Primary	4 115	28.4	17.9	(16.4, 19.0)	1.053	(0.745, 1.488)
	Secondary	9 391	64.8	15.4	(14.6, 16.3)	0.891	(0.634, 1.252)
	Higher	714	4.9	12.6	(9.9, 15.3)	0.707	(0.468, 1.069)
Contraceptive	No method	8 076	56.1	14.4	(13.6, 15.3)	Ref	
	Traditional	107	0.7	21.0	(11.9, 30.0)	1.571	(0.909, 2.714)
	Modern	6 308	43.2	16.7	(16.6, 18.7)	1.269	(1.146, 1.406)
Religion	Apostolic	4 732	32.7	15.7	(14.4, 16.9)	Ref	
	Muslim	70	0.5	24.4	(14.5, 34.4)	1.748	(0.997, 3.046)
	None	2 035	14.0	17.2	(15.4, 19.1)	1.158	(0.993, 1.351)
	Other Christians	7 333	50.6	15.6	(14.7, 16.5)	1.005	(0.900, 1.121)
	Traditional	321	2.2	16.6	(12.3, 20.9)	1.073	(0.781, 1.476)
Believe Condom works	Don’t know	424	2.9	8.2	(5.1, 11.3)	Ref	
	No	1 877	13.0	12.3	(10.7, 13.9)	1.574	(1.019, 2.433)
	Yes	12 190	84.1	16.8	(16.0, 17.6)	2.260	(1.500, 3.405)
Recent sex activities	Never had sex	2 872	19.8	4.3	(3.5, 5.1)	Ref	
	Not active last month	3 680	25.4	22.2	(20.7, 23.7)	6.397	(5.211, 7.851)
	Postpartum	519	3.6	19.1	(15.5, 22.7)	5.268	(3.901, 7.112)
	Currently active	7 420	51.2	17.0	(16.0, 18.0)	4.584	(3.753, 5.598)

^1^Literacy was measured in terms of ability to read and write. Non-literate: those who cannot read nor write; Partially: those who can read or write part of a sentence; Literate: those who can read and write a full sentence.

^2^Wealth Index is a composite measure of the household’s cumulative living standards based on a household’s ownership of selected assets such as television, vehicles as well as water access and sanitation facilities. It is generated using the principal component analysis and categorizes households into five wealth quintiles.

^3^Estimate is the HIV prevalence as a percentage.

**Fig 1:**
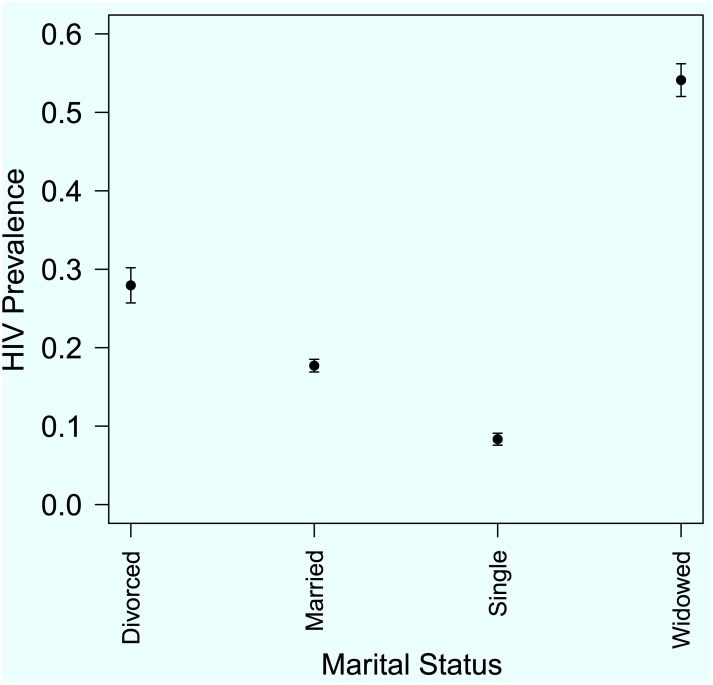
HIV prevalence rates across different categories of marital status.

**Fig 2:**
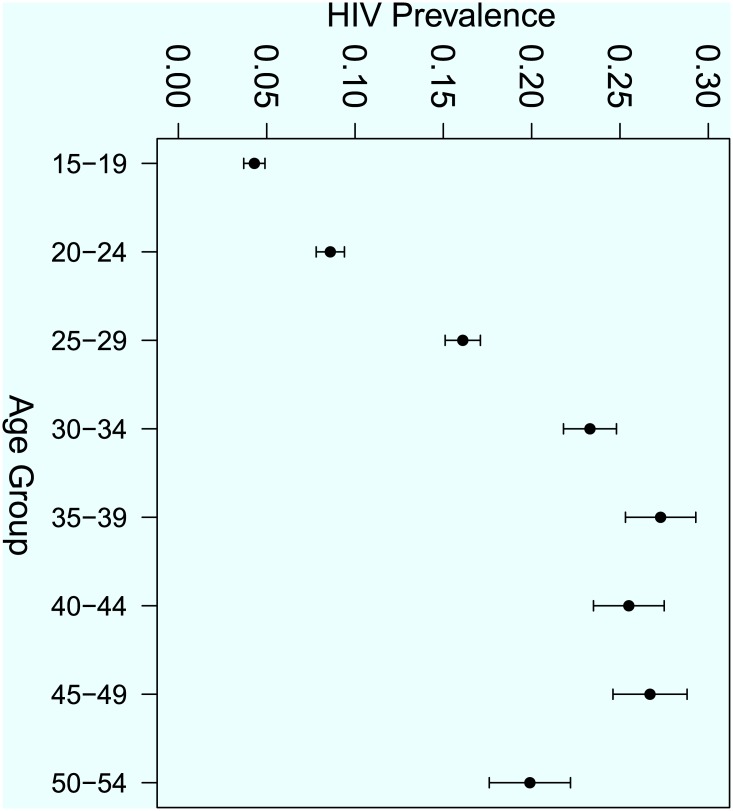
HIV prevalence rates across different categories of five year age-groups.

**Fig 3:**
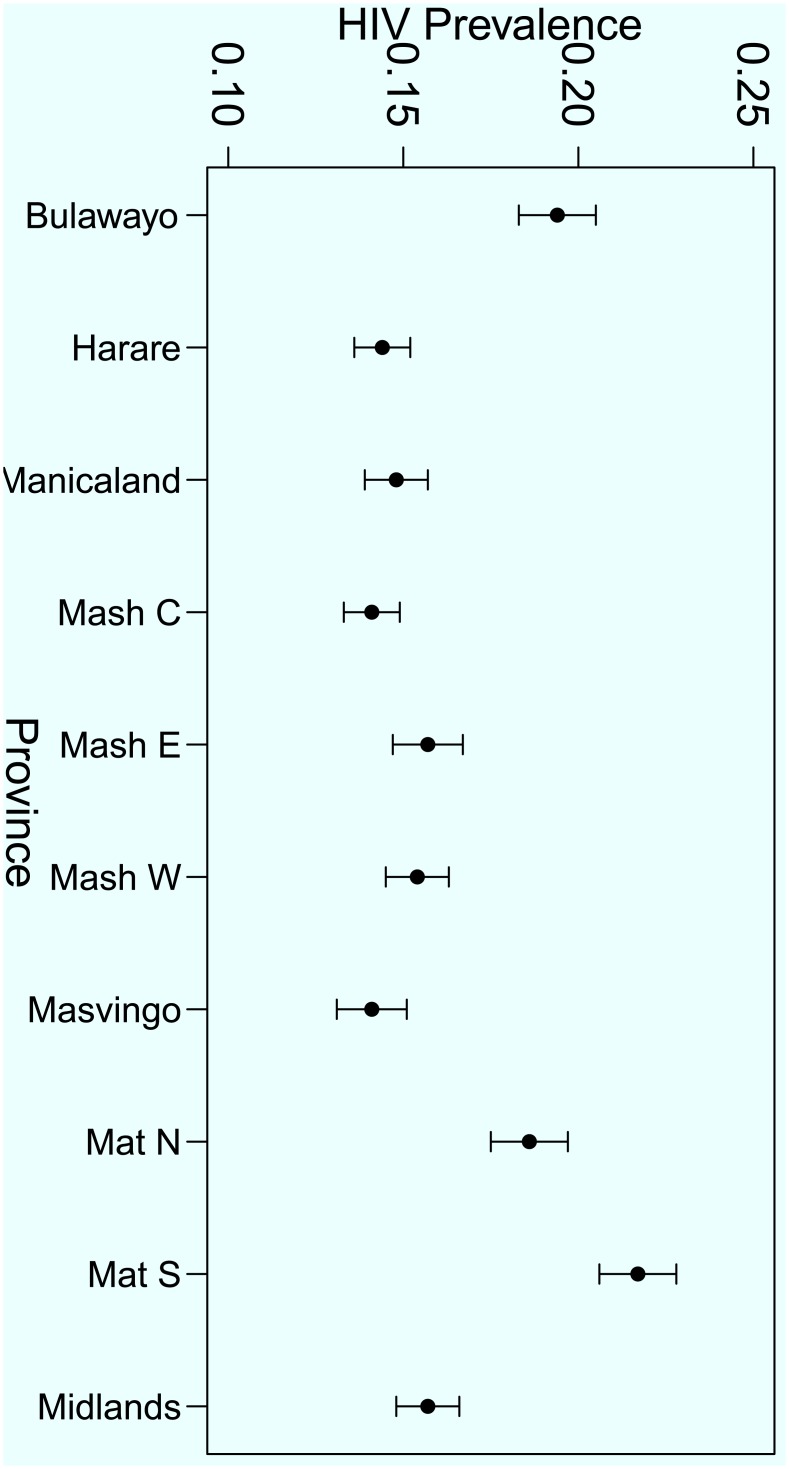
HIV prevalence rates in the different administrative provinces of the country.

In order to complement the descriptive statistics in Table 1, graphical presentation of the HIV prevalence were constructed for different domains in selected factors. For illustration purposes Figs 1, 2 and 3 show the variation of HIV prevalence across different categories of marital status, five-year age-groups and across the administrative provinces of the country respectively. The results show that there are significant differences in the HIV prevalence rates between the singles, the married, the divorced and the widowed. The highest HIV prevalence by marital status was among the widowed, ($\hat{p}=54.4\%,$ 95% *CI* = 51.3–60.0%) and the lowest was among the single/never married individuals ($\hat{p}=5.6\%,$ 95% *CI* = 5.2–6.6%). The results also show that, for five-year age-group the highest prevalence is among the 35–39 years age-group ($\hat{p}=26.9\%,$ 95% *CI* = 24.5–29.2) and the lowest rate is among the 15–19 years age-group $\hat{p}=4.0\%,$ 95% *CI* = 3.2–4.7%).

Other notable variations can be observed for the place of residence variable where HIV prevalence is significantly higher among the urban dwellers ($\hat{p}=16.8\%,$ 95% *CI* = 15.7–18.0%) than for the rural residents ($\hat{p}=14.7\%,$ 95% *CI* = 13.9–15.4%). Regarding the recent sexual activity variable, those that never had sex have a significantly lower HIV prevalence ($\hat{p}=4.3\%,$ 95% *CI* = 3.5–5.1%) than the other categories, and those that were not sexually active in the previous month have the highest prevalence rate ($\hat{p}=22.2\%,$ 95% *CI* = 20.7–23.7%).

For the variation across provinces as shown in Fig 3, Matabeleland South province has the highest rate ($\hat{p}=21.1\%,$ 95% *CI* = 19.5–24.0%) whereas Mashonaland Central province has the lowest ($\hat{p}=13.8\%,$ 95% *CI* = 12.5–15.8%). Matabeleland North province ($\hat{p}=18.5\%,$ 95% *CI* = 14.8–22.2%) and Bulawayo ($\hat{p}=18.8\%,$ 95% *CI* = 15.9–21.8%) also have relatively higher HIV prevalence than the rest of the provinces whose HIV prevalence rates range between $\hat{p}=13.6\%$ to $\hat{p}=15.6\%$. Table 1 also displays crude (unadjusted) *OR*s for each risk factor to quantify the likelihood of being HIV positive for each category relative to a reference level. For instance, for the factor gender, relative to the females, the males have a lower risk (*OR* = 0.69 95% *CI* = 0.624–0.763%) of being HIV positive. For the marital status factor, with reference to those who are single/never married, it is over three times more likely (*OR* = 3.224, 95% *CI* = 2.796–3.718%) for the married individuals, over six times more likely for divorced individuals (*OR* = 6.511 95% *CI* = 3.495–5.439%) and almost twenty times more likely (*OR* = 19.94, 95% *CI* = 16.038–24.791%) for widowed individuals to be HIV positive. All the other ORs in Table 1 can be interpreted in a similar way.

### 2.2 Logistic Regression Analysis

**Fig 4:**
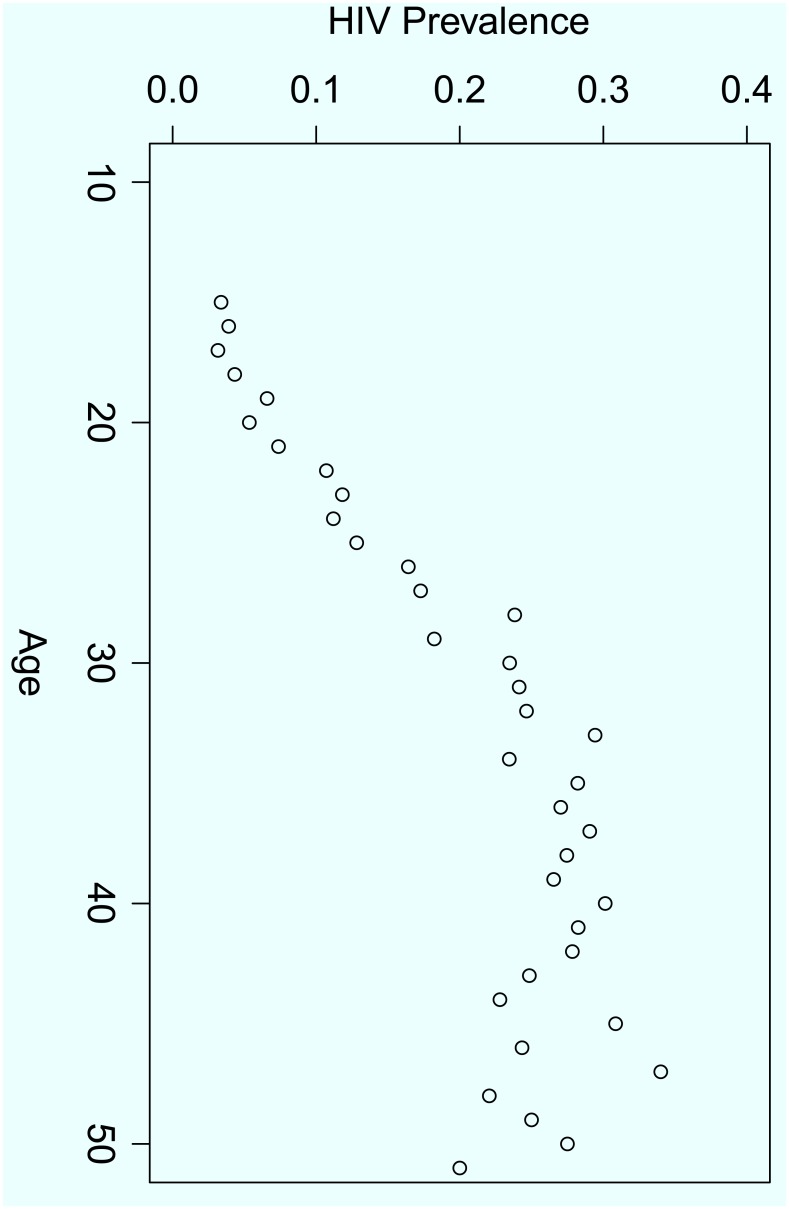
The average HIV prevalence per a given age versus age.

This section presents details of how the logistic regression analysis was used to construct a model for HIV prevalence. For the preliminaries we considered the relationship between HIV prevalence and age as a continuous variable. Fig 4 gives a plot of the average HIV prevalence rate for a given age against age. It is evident that there is generally a positive linear relationship with some curvature among the old respondents.

Bivariate design-based tests for association between each of the categorical predictor variables and the response variable were also performed. In particular the Rao-Scott test by [27] that takes account of the design-induced distortion of the asymptotic distribution of the Pearson and the likelihood ratio statistics was used. Table 2 displays the test results. The results indicate that the factors religion and wealth index are not significantly associated with HIV whereas the rest of the factors are significantly associated based on the p-values. The non-significant variables were not dropped completely for the computations of the multivariable survey logistic regression model, however even after being included they were found to also contribute insignificantly in explaining the variation in HIV.

**Table 2:** Rao-Scott (*F*-based) test statistics and p-values, for association of individual predictor variables and HIV status.

Variable	Levels	*n*	Percentages	HIV positive	*F-*value	*p-*value
Gender	Female	8 169	56.4	1 521	82.92	<0.001
	Male	6 322	43.6	867		
Marital Status	Single	4 777	33.0	308	326.17	<0.001
	Married	8 226	56.8	1 465		
	Divorced	877	6.1	277		
	Widowed	611	4.2	338		
Age Group	15–19	3 295	22.7	140	109.06	<0.001
	20–24	2 749	19.0	252		
	25–29	2 522	17.4	443		
	30–34	1 961	13.5	490		
	35–39	1 601	11.0	444		
	40–44	1 138	7.9	310		
	45–49	892	6.2	244		
	50–54	333	2.3	65		
Employment	Yes	6 591	45.5	1 216	30.27	<0.001
	No	7 900	54.5	1 172		
Place of Residence Education	Rural	9 839	67.9	1 551	2.29	0.0220
	Urban	4 652	32.1	837		
	No Education	271	1.9	48	5.80	0.001
	Primary	4 115	28.4	774		
	Secondary	9 391	64.8	1 475		
	Higher	714	4.9	91		
Wealth Index	Poorest	2 811	19.4	483	1.41	0.230
	Poorer	2 652	18.3	430		
	Middle	2 742	18.9	475		
	Richer	3 134	21.6	549		
	Richest	3 152	21.8	451		
Literacy	Non-literate	859	5.9	133	7.29	0.001
	Partially	1 103	7.6	229		
	Literate	12 529	86.5	2 026		
Religion	Apostolic	4 732	32.7	754	1.96	0.099
	Muslim	70	0.5	20		
	Non	2 035	14.0	356		
	Other Christians	7 333	50.6	1 197		
	Traditional	321	2.2	61		
Contraceptive	Non	8 076	56.1	1 246	10.82	<0.001
	Traditional	107	0.7	19		
	Modern	6 308	43.2	1 155		
Believe condom works	Don’t know	424	2.9	37	17.55	<0.001
	No	1 877	13.0	228		
	Yes	12 190	84.1	2 146		
Recent sex activities	Never had sex	2 872	19.8	128	121.45	<0.001
	Not active last month	3 680	25.4	846		
	Postpartum	519	3.6	106		
	Currently active	7 420	51.2	1 329		

**Fig 5:**
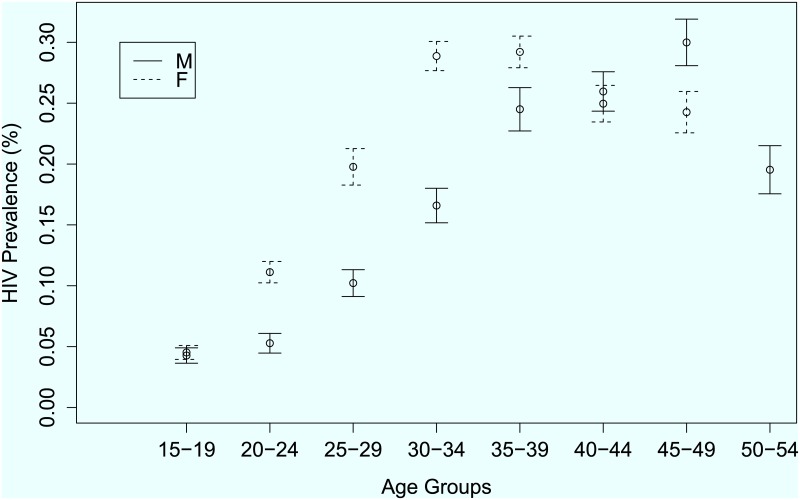
HIV prevalence estimates and their respective 95% confidence intervals by age group for males and females separately.

We established that HIV prevalence varies considerably with gender for each age group as displayed in Fig 5. The plot shows that although HIV prevalence generally increases with age for both males and females, it rises faster in females than in males among the lower age groups, however the prevalence becomes higher among males than among females from the 40–44 year age group and older. This implies that the risk of HIV varies by gender across different age groups necessitating the inclusion of a gender by age group interaction effect.

From a logistic regression modelling perspective, the results show that HIV prevalence is dependent on gender, age, marital status, literacy level, place of residence, recent sexual activities, one's opinion on whether condom use helps reduce risk of contracting HIV and a gender by age interaction effect (estimates of the model not shown here). The H-L goodness of fit statistic $\hat{C}=15.157$ on *g* − 2 = 8 d.f giving a p-value of 0.141 showing no evidence of lack of fit.

In order to facilitate the interpretation of the computed model, we expressed the parameter estimates as adjusted (to control for the effect of other factors in the model) ORs and assess the effects of confounding. It is important to note that in the presence of confounding among the variables, the adjusted ORs take precedence. We presented the crude ORs for the factors included in the final model. The results are displayed in Table 3. For the interpretation of the ORs we assume the reference cell method as explained by [26]—[28].

**Table 3:** Estimated adjusted ORs together with their respective 95% confidence intervals for the parameter estimates for the logistic regression model.

Parameter	Crude OR	95% CI	Adjusted *OR*	95% CI
Intercept			0.024	(0.016, 0.036)
Gender				
Male	0.696	(0.624, 0.763)	0.992	(0.665, 1.503)
Age Group				
20–24	2.109	(1.664, 2.673)	1.944	(1.386, 2.726)
25–29	4.360	(3.495, 5.439)	3.454	(2.487, 4.797)
30–34	7.159	(5.756, 8.902)	5.211	(3.739, 7.262)
35–39	8.556	(6.848, 10.690)	5.098	(3.626, 7.166)
40–44	7.865	(6.210, 9.959)	3.041	(2.082, 4.440)
45–49	8.427	(6.579, 10.793)	2.914	(1.939, 4.379)
50–54	5.572	(3.919, 7.921)	3.349	(2.050, 5.470)
Marital Status				
Married	3.224	(2.796, 3.718)	0.922	(0.727, 1.169)
Divorced	6.511	(5.320, 7.967)	1.801	(1.374, 2.359)
Widowed	19.940	(16.038, 24.791)	4.484	(3.315, 6.067)
Literacy				
Partially	1.489	(1.153, 1.924)	1.536	(1.145, 2.061)
Literate	1.083	(0.884, 1.326)	1.240	(0.982, 1.567)
Place of Residence				
Urban	1.184	(1.069, 1.312)	1.225	(1.087, 1.381)
Recent Sex Activities				
Not active last month	6.397	(5.211, 7.851)	2.112	(1.651, 2.701)
Postpartum	5.268	(3.901, 7.112)	1.707	(1.200, 2.428)
Currently active	4.584	(3.753, 5.598)	1.826	(1.405, 2.373)
Believe condom works				
Don’t know	1.574	(1.019, 2.433)	0.575	(0.386, 0.855)
No	2.260	(1.500, 3.405)	1.136	(0.964, 1.338)
Age group*Gender				
20–24: Male			0.462	(0.263, 0.797)
25–29: Male			0.484	(0.295, 0.792)
30–34: Male			0.550	(0.339, 0.894)
35–39: Male			0.919	(0.565, 1.496)
40–44: Male			1.726	(1.019, 2.922)
45–49: Male			2.289	(1.305, 4.015)

The results in Table 3 show that the females are slightly more at risk (*OR* = 0.992, 95% *CI* = 0.665–1.503%) of being HIV positive than the males, holding the effects of the other variables in the model constant. With reference to the 15–19 year old respondents, the results show that the risk of being HIV positive increases with age, peaking among the 30–34 year old respondents (*OR* = 5.211, 95% *CI* = 3.739–7.262%) before falling among the ‘older' respondents, controlling for the other variables in the model. Relative to the single/never married, the results show that the risk of being HIV positive is slightly lower (*OR* = 0.922, 95% *CI* = 0.727–1.169%) among the married, almost twice (*OR* = 1.801, 95% *CI* = 1.374–2.359%) among the divorced and over four times (OR = 4.484, 95% *CI* = 3.315–6.067%) among the widowed controlling for the effect of the other variables in the model.

With reference to the non-literate, the partially literate are approximately one and a half (*OR* = 1.536, 95% *CI* = 1.15–2.061%) more likely and the literate are slightly more likely (*OR* = 1.240, 95% 0.982 − 1.567) to be HIV positive controlling for the effect of the other variables in the model. In relation to those respondents who have never had sex, those who were not sexually active (for reasons other than postpartum) in the previous four weeks were over twice as likely (*OR* = 2.112, 95% *CI* = 1.651–2.701%), whereas those who were not sexually active (for postpartum reasons) in the previous four weeks were more likely (*OR* = 1.707, 95% *CI* = 1.200–2.428) and those who were currently sexually active were almost twice as likely (*OR* = 1.826, 95% *CI* = 10405–2.373) to be HIV positive. Regarding individuals' opinion on whether condom use reduces the risk of contracting HIV, the chances of being HIV positive is less (*OR* = 0.575, 95% *CI* = 0.386–0.855) among those who say they do not know and the chances are slightly more (*OR* = 1.136, 95% *CI* = 0.964–1.338) among those who say no relative to those who answered yes, holding other variables in the model constant.

The five-year age-group by gender interaction (effect modification) shows the additional effect of age on HIV prevalence for males in relation to females. The results show that among the lower age-groups (20–24, 25–29 and 30–34) age has an increasing effect on HIV prevalence for females as compared to the males whereas among the older age-groups, age has an increasing effect on HIV prevalence for males as compared to the females. This implies that it is more likely for a young female person to be HIV positive as compared to a young male person, which is commensurate with a general belief that young women engage in sexual activities with older men.

## Discussion

There are a variety of possible reasons for the observed variations in HIV prevalence across various categories of the risk factors. These are mainly driven by biological, socio-economic and socio-cultural factors. It is important to note that sexual contact remains the key driver of HIV transmission among the sexually active population in sub-Saharan Africa. In particular the higher risk of HIV among the females as compared to the males could be explained by a number of factors that make females more vulnerable. These include different rates of sexual interaction and relationships between males and females brought about by socio-cultural practices such as polygamy that give rise to differing susceptible rates to HIV. In addition, socio-economic issues such as economic dependence of women on men, that is still highly prevalent in most sub-Saharan African countries, leaves women with limited negotiating power with regards to sexual matters putting them at more risk. It is worth noting that during penile-vaginal intercourse, a woman's body is more susceptible to HIV infection than a man's. There is increased surface area of the body parts of a woman where HIV transmission can occur than on a man.

The relatively low prevalence among the single/never married individuals possibly points to the fact that HIV transmission is mainly driven by sexual contact, and as such most of those who are single/never married are most likely not yet sexually active. On the other hand the relatively high prevalence among the widowed may be a indication that the partner lost died due to AIDS. Similar to the variation across age groups, the relatively low prevalence among the 15–19 is perhaps due to the fact that these are mainly young, possibly of school going age and relatively less sexually active. On the contrary the middle ages, 25–40 year old are often characterized by high sexual activities hence a possible explanation for the relatively high prevalence.

The observed urban-rural variation can be attributed to the fact that majority of urban residents are middle aged and are synonymous with relatively high sexual activities. In addition, commercial sex activities are also prominent in urban areas than in rural areas, argued as one of the key contributors of new HIV infections. The lack of sexual activity reported among those with high prevalence is possibly linked to their HIV status. Studies have shown that although reaction to the discovery of one’s seropositivity varies, there is a general decline in sexual activity. The significantly high HIV prevalence in Matabeleland South province is possibly due to the border towns in the province, such as Beitbridge and Plumtree that are synonymous with increased commercial sex activities.

The additional effect of age on the gender effect on HIV prevalence that sees the young females being at higher risk of HIV than their young male counterparts agrees well with a general belief that young females engage in sexual activities with older males. From an epidemiological perspective, the variable gender is considered an effect modifier on age group as a risk factor in explaining the variation in HIV. There is generally a similar trend in the variation in HIV prevalence across different categories of the risk factors between the adjusted and the crude ORs except in the magnitude of the risk across the categories.

## Potential Strengths and Limitations of Study

The study draws its main strength from the appropriate application of sound statistical methods coupled with the utilization of advanced statistical computing software in estimating HIV prevalence. However, a potential drawback of the current study comes from the use of secondary data which often leaves the data analyst with limited control over the data collection process although this is not to downplay DHS data that are carefully collected by a team of highly trained statisticians with excellent expertise in survey methodology. Furthermore, the complete case analysis approach that we made regarding missing data often results in loss of statistical information especially if the distribution of observed data is different from that of the missing data. Thus the current study can be regarded as a base up on which future research on estimation of HIV prevalence using population-based data can be built.

## Conclusion

Estimating national HIV prevalence using data obtained from sampling a subgroup of the entire population is argued to have shortcomings in that the estimates might be biased especially if the subgroup is not representative of the target population. In addition the estimation does not display the variation across different domains of the population. Using population-based survey data supported by the use of sound statistical methodology for analyzing complex survey data can enhance better estimation of both national and domain level HIV prevalence. Furthermore, explaining variation in HIV using risk factors is possible since the use of population-based survey data allows linking HIV status to demographic, socio-economic, socio-cultural and behavioural factors, making use of the proximate determinants conceptual framework. The current study provided crude design-based estimates of HIV prevalence, at the national level as well as domain estimates based on the prominent risk factors in the population. From a modelling stand-point, a survey logistic regression model was used to provide a way of explaining the variation in HIV prevalence using socio-economic, socio-cultural, behavioural and demographic variables.

The results from the study showed that HIV prevalence is dependent on age, gender, marital status, literacy level, level of recent sexual activities, belief about whether condom use reduces risk of HIV and place of residence. The study also showed that one's religion, education level and wealth status do not play a significant role in determining one's HIV status. The study reveals that HIV prevalence is higher among females than in males (both crude and adjusted). Generally HIV prevalence increases with age. This shows that, for randomly selected ‘older' person, the probability of obtaining an HIV positive individual is higher than for a ‘younger' person. The results also showed that, as compared to the single or never married people, the married, divorced and widowed are more likely to be HIV positive. People in the urban areas have higher HIV prevalence as compared to their rural counterparts. The study also found that age has an increasing effect (effect modification) on gender regarding HIV prevalence in males than in females. That is, HIV prevalence increases, with age, at a faster rate in males than in females.
